# Biological Regulatory Network (BRN) Analysis and Molecular Docking Simulations to Probe the Modulation of IP_3_R Mediated Ca^2+^ Signaling in Cancer

**DOI:** 10.3390/genes12010034

**Published:** 2020-12-29

**Authors:** Humaira Ismatullah, Ishrat Jabeen, Muhammad Tariq Saeed

**Affiliations:** Research Center for Modeling and Simulation (RCMS), National University of Sciences and Technology (NUST), Academic-I Building, H-12 Islamabad 44000, Pakistan; hismatullah.phd15@rcms.nust.edu.pk (H.I.); tariq@rcms.nust.edu.pk (M.T.S.)

**Keywords:** IP_3_R, Ca^2+^ signaling, cell proliferation, apoptosis, oncogenes, tumor suppressors, biological regulatory network (BRN), molecular docking simulations, IP_3_R modulation

## Abstract

Inositol trisphosphate receptor (IP_3_R) mediated Ca^+2^ signaling is essential in determining the cell fate by regulating numerous cellular processes, including cell division and cell death. Despite extensive studies about the characterization of IP_3_R in cancer, the underlying molecular mechanism initiating the cell proliferation and apoptosis remained enigmatic. Moreover, in cancer, the modulation of IP_3_R in downstream signaling pathways, which control oncogenesis and cancer progression, is not well characterized. Here, we constructed a biological regulatory network (BRN), and describe the remodeling of IP_3_R mediated Ca^2+^ signaling as a central key that controls the cellular processes in cancer. Moreover, we summarize how the inhibition of IP_3_R affects the deregulated cell proliferation and cell death in cancer cells and results in the initiation of pro-survival responses in resistance of cell death in normal cells. Further, we also investigated the role of stereo-specificity of IP_3_ molecule and its analogs in binding with the IP_3_ receptor. Molecular docking simulations showed that the hydroxyl group at R_6_ position along with the phosphate group at R_5_ position in *‘R’* conformation is more favorable for IP_3_ interactions. Additionally, Arg-266 and Arg-510 showed π–π and hydrogen bond interactions and Ser-278 forms hydrogen bond interactions with the IP_3_ binding site. Thus, they are identified as crucial for the binding of antagonists.

## 1. Introduction

Calcium signaling in the intracellular environment plays a pivotal role in the plethora of cellular processes ranges from cardiac action potential to cell division, proliferation, metabolism, bio-energetics to autophagy, and apoptosis, thereby controlling cell fate [1,2,3,4]. The endoplasmic reticulum (ER) is the major intracellular Ca^2+^ storage organelle where intracellular Ca^2+^ accumulation is occurred by active transport mediated by Sarco/Endoplasmic Reticulum Ca^2+^ ATPases (SERCA) [5].

Under normal physiological conditions, a low intracellular concentration gradient of Ca^2+^ in ER, mitochondria, and cytosol as compared to the extracellular pool is maintained by a dynamic equilibrium between active uptake via Ca^2+^ pumps and release by Ca^2+^-release channels, the inositol 1,4,5-trisphosphate receptors (IP_3_Rs) [6]. Within a cell, Ca^2+^ binds with calmodulin (CaM) to regulate a variety of kinases and phosphatases, which further modulate cell proliferation and autophagy/apoptosis [7,8]. Therefore, any deregulation in intracellular Ca^2+^ homeostasis may disrupt the cell-cycle, which subsequently affects the ability of cells to regulate division through the cell-cycle or may lead to uncontrolled proliferation and tumorigenesis. Furthermore, IP_3_Rs mediated Ca^2+^ transfer from the ER to mitochondria has been recognized as crucial for overall Ca^2+^ homeostasis [6,9,10,11].

Briefly in non-excitable cells, the binding of extracellular signals (such as growth factors), facilitates phosphatidylinositol 4,5-bisphosphate (PIP_2_) cleavage by PLC (phospholipase enzyme C). This results in the production of lipid diacyl-glycerol (DAG) and an inositol 1,4,5 tris-phosphate (IP_3_) molecule as a soluble second messenger [12]. Subsequently, IP_3_ binds to its receptor (IP_3_R) present on the endoplasmic reticulum [13] where a low level of IP_3_ is responsible for IP_3_R channel activation in basal conditions. Additionally, several oncogenes and tumor suppressors (such as B-cell lymphoma 2 (Bcl-2) family proteins [14,15], IP_3_R-binding proteins [16], phosphatase and tensin homolog (PTEN) [17,18,19] and BRCA1 associated protein 1 (BAP1) [20,21]) directly bind with IP_3_Rs and modulate their activity to control Ca^2+^ influx into mitochondria. Under various pathophysiological conditions, changes in the expression level of these binding factors may alter the activation of IP_3_R, consequently affecting the mitochondrial Ca^2+^ concentration level followed by uncontrolled cell growth or cell death [16,21]. Therefore, a reduction of Ca^2+^ influx by non-covalent blockade of IP_3_Rs may reduce Ca^2+^ dependent cell proliferation and induce Ca^2+^ dependent autophagy or cell death. Inhibition of IP_3_R dependent calcium signals in malignant cells may also show impaired mitochondrial bioenergetics, activated AMP kinases, and autophagy [22].

Overall, three autonomous tissue-specific isoforms encoded by distinct genes (ITPR) of IP_3_R as type IP_3_R_1_, IP_3_R_2_, and IP_3_R_3_ have been identified so far. Moreover, these three isoforms share 60–80% homology, with higher similarity in the pore region and ligand-binding domain [12,23,24]. The three isoforms have been characterized by overlapping expression patterns with most of the cells expressing more than one isoform simultaneously [25,26,27]. However, in mammalian cells, IP_3_R_3_ is predominantly present in all cell types [26,28,29,30].

In the current study, we have modeled the general downstream signaling pathway of IP_3_R with proto-oncogenes and tumor suppressor genes to probe the impact of IP_3_R inhibition on Ca^2+^ signaling and subsequently cell proliferation and autophagy/apoptosis. Furthermore, the ligand–protein interaction profile of already known IP_3_ derivatives has been studied to understand the 3D structural features of the IP_3_R modulators.

## 2. Materials and Methods

### 2.1. Construction and Dynamic Simulations of Biological Regulatory Network (BRN)

A knowledge-driven biological regulatory network (BRN) was constructed by collecting a set of oncogenic and tumor suppressor proteins interacting (either activating or inhibiting) with IP_3_R from intensive literature search [31,32,33,34] and Kyoto Encyclopedia of Genes and Genomes (KEGG) database [35]. For the construction of a biological regulatory network (BRN), only those modulatory proteins were selected that directly or indirectly interact with IP_3_R (Supplementary Table S1). In BRN, the proteins were represented as nodes and the distinct interaction types (activation & inhibition) between these proteins were represented as a directed edge connecting both nodes. The graphical representation of the biological regulatory network (BRN) was prepared using an interactive graphical software yEd v3.18.1.1 [36].

Initially, a discrete biological regulatory network model was developed by defining parameters 0.0 and 1.0 for inactive and active states respectively for an entity in the network. This is followed by continuous modeling and dynamic simulation of the network to probe the role of IP_3_R mediated Ca^+2^ signaling in cell proliferation and apoptosis under three different conditions (a) in normal cells, (b) in cancer cells and (c) in the presence of IP_3_R’s inhibitors.Java genetic regulatory network simulation framework (Jimena) software [37] was used to interpolate activation and inhibition states of different nodes defined by Boolean algorithm [38]. Overall, the simulation of constructed biological regulatory network (BRN) was performed by continuous modeling, where nodes have values between the interval (0, 1), and real-valued ODEs (ordinary differential equations) determine the behavior of the network. During simulations, at each step of Boolean expressions, the parameter values were updated by built-in updating schematic function known as asynchronous random Boolean networks (ARBN) [39]. The value of each state is updated as a function of state value relative to the time of the previous node, with which it has an incoming direct link. Further, each node calculates its next state from the parental node or incoming direct neighboring node by deterministic Boolean function [39,40]. Also, value of the next node is decided randomly, and nodes are considered heterogeneous with no spatial network pattern. As a result, the adjacency matrix in a given network topology is calculated randomly depending upon the state of each node, either constant or stochastically changing [40].

The dynamic behavior of the network was further analyzed by performing the simulation of the network using the SQUAD simulation method [41]. Briefly, SQUAD works on the principle of binary decision diagram algorithm considering the network as a discrete dynamical system first and then localizes the steady states of the network followed by the creation of a continuous dynamical biological regulatory network [41]. In the constructed BRN, the node (proteins) values were normalized-Hill functions as explained by Wittmann et al. [42] with SQUAD decay as 1.0 and SQUAD steepness as 10 [41,43]. The network was simulated for 400 simulation-time seconds with the step size (dt) defined as 0.05. The data, obtained from simulations, was imported into an open-source interactive graphical tool plotly [44] (https://plot.ly/) to generate graphs where default axes settings were used to probe the dynamical behavior of the selected entities in the IP_3_R regulatory network.

### 2.2. Ligand Data Collection & Rectification

A dataset (Supplementary excel file) of already known 40 inositol analogs, cyclic quinolizidines, and other structurally diverse compounds, with known inhibitory potency (IC_50_) values against IP_3_R was collected from literature [31,45,46,47,48,49] and from the ChEMBL database [50]. The dataset includes only those competitive inhibitors of IP_3_ binding for which inhibitory potency against IP_3_R was calculated by fluorescence assay [51,52]. Additionally, the stereochemistry of each stereoisomer was manually corrected and redrawn by Marvin Sketch version 18.8 [53]. Molecular Operating Environment (MOE) version 2019.01 [54] was used to generate 3D structures of all compounds followed by protonation, partial charges calculation, and energy minimization using MMFF94x force field [55].

### 2.3. Molecular Docking Simulations & Pose Analysis

The recent availability of EM structural details of IP_3_R_3_ of human [54] in apostate, ligand-bound state, and with calcium ion bounded, paved the way to elucidate the binding mode of IP_3_ receptor modulators. IP_3_R_3_ EM structure in the apostate [PDB ID: 6DQJ with 3.49Å resolution] was selected for molecular docking simulations of the whole data set of IP_3_R modulators (Supplementary excel file). Monomeric form (chain A) of protein was selected and prepared for molecular docking simulations by protonating first at pH 7.4, followed by the energy minimization using AMBER99 force field [56,57] in MOE 2019.01 [52]. Binding coordinates of the active site of receptor protein were defined around 4.5 Å within IP_3_ binding domain [54] and from previous mutagenesis knowledge [57,58]. Overall, the important amino acid residues in the active site of the IP_3_R_3_ including Arg-266, Thr-267, Thr-268, Leu-269, Arg-270, Arg-503, Glu-504, Arg-505, Leu-508, Arg-510, Glu-511, Tyr-567, and Lys-569 were selected as a binding pocket. Briefly, induced-fit docking protocol was selected to generate 100 binding solutions per ligand using default Alpha Triangle as placement method and Alpha HB as a scoring function [52]. The ligands were considered flexible. However, associated stereochemistry was kept fixed. Overall, the binding scores of the top 10 binding solutions per ligand were correlated with the biological activity (pIC_50_) values. Finally, one binding solution per ligand, which provides a good correlation (R^2^) with the biological activity value (pIC_50_), was selected for further protein-ligand interaction profiling (PLIF).

## 3. Results

### 3.1. Biological Regulatory Network (BRN) Simulations of IP_3_R

**Figure 1:**
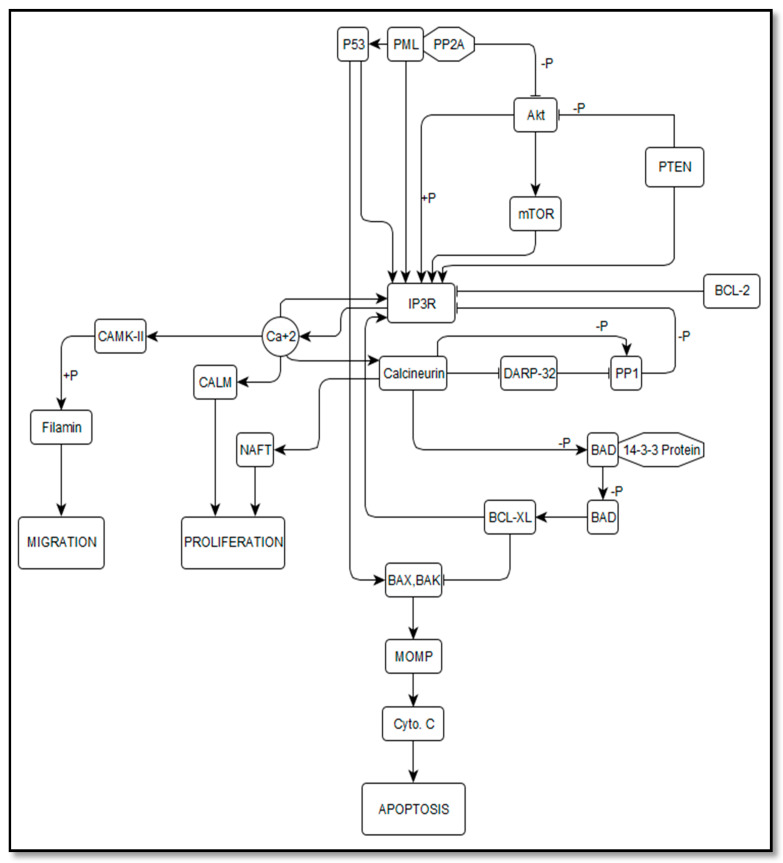
Biological regulatory network (BRN) of IP_3_R with tumor suppressor and onco-proteins. The IP_3_ mediated Ca^+2^ signals play an important role in cell proliferation via the Nuclear Factor of Activated T-cells (NFAT) pathway triggered by Calcineurin. It also mediates cell migration by activating Ca^2+^/CaM-dependent phosphatase and Ca^2+^/CaM-kinases (CaMK). Whereas upon inhibition of IP_3_R, the activated Calcineurin also triggers the downstream apoptotic pathway by de-phosphorylating BAD that results in activation of members of the Bcl-2/Bax family.

The tumor suppressors and proto-oncogenes interacting with IP_3_R were selected for the construction of knowledge-driven theoretical biological network (BRN) as shown in Figure 1. Cell proliferation and autophagy/apoptosis were selected as simulation responses. The downstream signaling cascades of Ca^2+^/CaM-dependent phosphatase and Ca^2+^/CaM-kinases (CaMK) were considered in the case of cell proliferation. Additionally, calcineurin (CaN) one of the distinctive downstream targets, which triggers the nuclear factor of activated T-cells (NFAT) pathway [58,59,60], favoring the nuclear translocation of dephosphorylated NFAT1 molecule and triggers the cell to proceed into S-phase [61,62,63] was also considered for BRN construction. Overall, the Ca^2+^/CaM-kinases (CaMK) pathway has been considered important in the progression of the cell cycle, while the Ca^2+^/calcineurin (CaN) pathway; not only regulates the checkpoints of the cell cycle but also plays a crucial role in the transition of the cell cycle between G1 and S phases [60,64,65].

Moreover, manipulation of Ca^2+^ signaling from the endoplasmic reticulum to mitochondria via members of the Bcl-2/Bax family has been considered crucial in the regulation of autophagy leading towards cell death [66,67]. Furthermore, in the initial phase of cell death (autophagic or apoptotic), IP_3_R has been recognized as an important contender [68]. Thus, IP_3_R mediated Ca^2+^ signals not only regulate the cell cycle but also inflect the decision of cell survival or cell death [69]. Detailed information of selected gene products and their physiological functions has been provided in the supporting information (Table S1).

Briefly in the network, the assumption of IP_3_R node as a stable state (SS) is imperative, where every node has a zero concentration parameter value indicating the system prior to any perturbation. Within the network, IP_3_R was assumed as the central node and a key component responsible for an influx of Ca^2+^ signals into the mitochondria as shown in Figure 1. Moreover, IP_3_R was considered as the initiator of the network, followed by the subsequent dynamic simulations of continuous modeling. The initial parameters for scaled concentration values were defined 0.0 as inactive states, 0.1–0.4 as low activation state, 0.5–0.9 as active, and 1.0 as hyperactive state in the BRN. Therefore, in order to correctly estimate the activation and inhibition relationship between the extracted nodes, the BRN of IP_3_R was simulated up to the time where all states become stable. Under normal cell dynamic simulations, the initial scaled concentration of IP_3_R and calcium was considered in low activation range (0), while to simulate the attenuated IP_3_R, a perturbation function was added and a moderately active state (0.5) was perused (Table 1). Whereas, in cancerous model dynamic simulations, the initial concentration of IP_3_R was considered as hyperactive, i.e., =1.0 as shown in Table 1.

#### 3.1.1. Dynamic Model Simulation in Normal Cells

**Figure 2:**
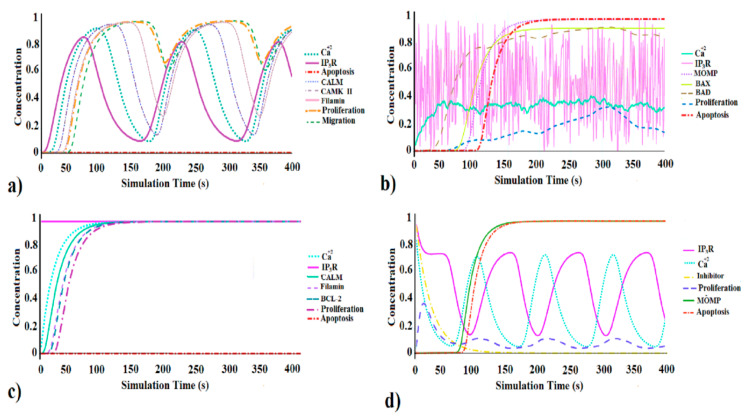
Dynamic simulations of the biological regulatory network of IP_3_R by Jimena showing the effect of IP_3_R mediated Ca^+2^ effluxes in the regulation of cell proliferation and apoptosis. The time (s) is shown on the *x*-axis and the relative activity of oncogenes and tumor suppressors, in terms of concentration, is shown on the *y*-axis. (**a**) Simulation results of normal IP_3_R function and its impact on cell growth & apoptosis. (**b**) Simulation results of perturbed IP_3_R in normal cell and resultant apoptosis and cell proliferation. (**c**) Simulation of highly active IP_3_R in the cancer cell. (**d**) Dynamic simulation results of IP_3_R network in a cancer cell in presence of inhibitor showing the effect of inhibitors upon cell proliferation and apoptosis.

During the simulation time, the calcium efflux was initiated due to IP_3_R activation, and a gradual increase in the Ca^+2^ at each time step was observed, which finally reached the maximum expression level (0.926) as shown in Figure 2a. This calcium is used to derive the bioenergetics of mitochondria [70] in terms of rate-limiting factor for production of ATP in the Krebs cycle [12,22]. The downstream cell cycle proliferation cascade was initiated by the activated calcium signals as shown in Supplementary Figure S2. Assuming the prior activation of IP_3_R signals (due to the binding of IP_3_ with its receptor) in the BRN, the signal peak of IP_3_R gradually reached its maximum expression level (0.945), which in turn initiated the calcium signaling. Figure 2a represents the biological expression of IP_3_R and calcium where significant activation of cell proliferation (0.956) and cell migration (0.931) was observed. Initialization of Ca^2+^ signals at ~30 s and subsequent activation of CALM and CAMK-II was observed that activated the cell proliferation (orange line) and cell migration (green line) nodes (Figure 2a). Both the pathways, Ca^2+^/CaM-kinases (CaMK) and Ca^2+^/calcineurin (CaN) have been considered crucial in cell cycle progression in terms of the cell cycle regulation and transition between different phases [60,64,65]. Taking into account the normal physiological conditions of the cell, when IP_3_R (0.945) and calcium signaling (0.926) are functioning properly, the nodes like CALM (0.929) and CAMK-II (0.929) were active, which are responsible for the activation of downstream cell proliferation cascade. Similarly, the cell proliferation (0.956) and cell migration (0.931) nodes were also observed as active and functional. Meanwhile, the expression of the apoptosis node (red line) was observed as inactive (0.0) during the entire simulation time (Figure 2a).

Next, the biological regulatory network (BRN) was tested for random activation values of the IP_3_R node by adding the perturbation function of Jimena over the entire simulation time. These perturbations are translated by Jimena into time-resolved activity curves and their effect is calculated at every single node of the regulatory network. The activation curves in Figure 2b indicate that the attenuation of IP_3_R leads toward the convoluted calcium oscillation signals (green line). Under normal physiological cell conditions, intrusion in the IP_3_R mediated calcium release or inadequate Ca^+2^ signals from ER to mitochondria creates stress and activates pro-survival responses (i.e., autophagy) [70,71]. However, cell survival response (autophagy) may lead to cell death if IP_3_R fails to regain its normal function or attenuation lasts for a prolonged time. The initial value of IP_3_R was set as moderately active (0.5) and during perturbed simulation time, the IP_3_R value decreased gradually from an initial active state to a low active state (0.393) as shown in Table 1. This decrease in IP_3_R value leads toward the subsequent drop (0.333) of calcium signals (cyan line in Figure 2b) compared to the normal cell conditions where calcium signals were active (0.926, Table 1). Similarly, a decrease in cell proliferation rate (blue line in Figure 2b) was observed i.e., from the active state (0.9) with normal cell condition to least active or nearly inactive (0.14, as per Table 1) state with attenuated IP_3_R. Moreover, the failure of IP_3_R to perpetuate its normal pool and to maintain a certain calcium level in the cell may provoke autophagy response in the cell. After 50 s, due to attenuated IP_3_R and decreased calcium concentration (0.333), it was observed that the BAD node (Figure 2b) was activated and gradually reached its maximum concentration expression level (0.994) as tabulated in Table 1.

Finally, instead of activation of cell proliferation signals as seen in normal cell conditions (Figure 2a), the activation of BAD node eventually activated the autophagic cascade of the biological regulatory network (BRN) leading towards cell death (apoptosis) response, initiated by the induction (0.997) of MOMP (mitochondrial outer membrane permeabilization) followed by the release of cytochrome c (Figure 2b). Previously, it was reported that the aberrant cell cycle activates the intrinsic apoptotic pathways that initiate mitochondrial membrane permeabilization (MMP), a process fairly regulated by Ca^2+^ signaling [72,73]. In Figure 2b, at a simulation time of 40 s, the activation of the BAD signal (0.994) resulted in the down-regulation of the cell proliferation (0.141). Consequently, the BAX protein signal originated and reached its maximum expression level (0.93, Table 1). The autophagic/apoptotic checkpoints of the mitochondria are controlled by the BCL-2 protein family, where BAK and BAX are considered crucial effectors of MOMP induction [74,75]. Here, deregulation of the cell cycle leads toward the initiation of intrinsic apoptotic mechanism regulated by p53, a tumor suppressor gene [76]. During the apoptosis cascade regulated by p53, cytochrome c is released from the mitochondria to the cytosol by the BAX and BAK [77]. Thereafter, in the initial phase of autophagic cell death, where IP_3_R was perturbed for a long time (entire simulation), the attenuated Ca^2+^ signals inflect the decision of cell death/(autophagy) [69] and eventually apoptosis (red line) curve initiated after 100 s of simulation times (Figure 2b).

#### 3.1.2. Dynamic Model Simulation in Cancer Cells

Cancer cells harness the calcium machinery of the host cell to proliferate at a maximum level and reduced apoptotic response by relying upon the high level of Bcl-2 [78]. The BRN was simulated with default values of decay Odefy [43] and SQAUD steepness [37,41]. However, to mimic the cancerous behavior of the model virtually, the initial parametric concentration value of the IP_3_R was set to maximum (1.0) as shown in Table 1. During the entire simulation time, the IP_3_R level abided super active (1.0) that subsequently activated the calcium signal curve (Figure 2c). Comparative to the normal cell conditions, where calcium signal oscillation repeats after regular intervals (Figure 2a), the expression of calcium signal curve reached its maximum (scaled) concentration level after initiation and remained super active (0.99) throughout the simulation as shown in Table 1. The Bcl-2 level (blue dotted line) initiated and gradually reached its maximum concentration level as shown in Figure 2c. The altered role of the Bcl-2 has been reported in many carcinomas [79]. In different cell types, it has a central role in regulating the apoptotic pathway by delaying or halting it [80]. Furthermore, members of Bcl-2 family proteins anchor the outer membrane of the mitochondria facing towards the cytosol [81]. One of the pro-apoptotic proteins, BAD dimerizes hetero-dynamically with the BCL-_XL_. This dimerization dissociates upon phosphorylation of BAD and abrogates its pro-apoptotic effect, hence; induces cell death [82].

In Figure 2c, the super activated calcium signals provoke the downstream CALM signals of the proliferative pathway. At simulation time of almost ~30 s, the curve of CALM signal originated and reached its maximum expression level 1.0, whilst the IP_3_R and calcium signals were super active. Considering the normal cell conditions, instead of oscillatory behavior of nodes, the values of the proliferative signal cascade (Ca^+2^, CALM, CAMK-II) of the biological regulatory network become constant (1.0) after 150 s. In contrast, the apoptotic signal (red line) was prolonged at its inactivation state (0.0, Table 1) throughout the simulation time as shown in Figure 2c. The activated level of IP_3_R is responsible for calcium signaling initiation that successively triggers the cell proliferation signals cascade. Previously, Davis et al. also related the elevated level of IP_3_R with cell proliferation, migration, and survival in cancer [83,84,85]. This further strengthens our observed correlation of calcium level in high cell proliferation and minimum apoptosis in cancer cells.

In normal cells, IP_3_R regulates autophagy as a pro-survival response, whereas, in cancer cells, IP_3_Rs are associated with metastasis [86]. Inhibition of IP_3_R results in compromised bioenergetics of cell, subsequently leading towards cell death. Considering the important role of inhibitors in cancer cells, a separate node of inhibitor was added in the “.graphml” file of yEd in the constructed biological regulatory network of IP_3_R (Supplementary Figure S2). Initially, the inhibitor was assumed as super active and the concentration was set as 1.0 to mimic the cancerous condition of the biological system. Considering the carcinomas condition, IP_3_R was also set as super active i.e., 1.0, whereas the initial concentration of calcium due to hyper-active IP_3_R was observed as 0.9 (Figure 2d). During the simulation time of 50 s, the concentration of inhibitor gradually dropped from hyperactive (1.0) to least active (0.15, Table 1). Moreover, in the presence of inhibitor, IP_3_R failed to maintain its normal pool. Hence, the concentration of IP_3_R also dropped from 1.0 to 0.75 and continued as constant till simulation time of 50 s. Afterward, the IP_3_R signal gradually dropped to low active state, whilst the calcium signals also dropped to the concentration of least active (0.15) level succeeding the pattern of inhibitor’s peak (Figure 2d).

The gradual drop in IP_3_R signal from super active to least active is opposite to the normal IP_3_R signal peaks where a steady increase was observed. This may be correlated with the effect of inhibitors in the biological system; the normal functionality of IP_3_R dropped, resulting in the delayed origination of calcium signal peaks concerning simulation time. Ultimately, the simultaneous decrease of IP_3_R and calcium signal peaks influenced the downstream proliferation pathway (blue dash line, Figure 2d). Initially, the proliferation signal was originated and reached to a moderate concentration level (0.4, Figure 2d), but, due to the decrease in calcium signaling it gradually decreased to almost an inactive state (0.148, Table 1). Comparative to the proliferation state of cancer cell (Figure 2c), where the proliferation signals remain active throughout the entire simulation time. This pattern of proliferation is might be due to the presence of inhibitor in the BRN. Similar to Figure 2b, the decrease in calcium signal resulted in a low proliferation rate (blue line) which ultimately triggered the apoptotic pathway (Figure 2d).

After 50 s of simulation time, when the concentrations of IP_3_R, calcium, and proliferation were 0.2, 0.15, and 0.16, respectively, the initiation of the MOMP signal was observed in the BRN as shown in (Figure 2d). Induction of MOMP signal is considered crucial as the first step of intrinsic apoptotic pathway. Initially, the MOMP signal was inactive for 65 s, but its activation stimulated the apoptotic signal in the BRN at ~85 s (Figure 2d), and consequently, the signal peak of apoptosis reached to the maximum level of concentration expression (1.0). The apoptotic cascade initiated early (~80 s) comparative to attenuated IP_3_R (Figure 2b) indicating the difference in pro-survival response (autophagy) of normal cells and importance of inhibitor in cancer cell for induction of apoptosis. This further necessitates probing the binding hypothesis of IP_3_R inhibitors to modulate activation of IP_3_R and Ca^+2^ signaling in cancer.

### 3.2. Data Set of IP_3_R Inhibitors and Structure Activity Relationship (SAR)

The already known data set of 40 inhibitors of IP_3_R is divided into two main classes namely class ‘A’ including (*myo*-inositol derivatives), and class ‘B’ (macro-cyclic oxaquinolizidine derivatives). Additionally, compounds belonging to structurally diverse scaffolds were classified as series M (miscellaneous). The biological activities of the whole data set vary from 0.0029–20,000 µM.

Class A consists of 12 compounds and based on the position and isomeric relationship of the substitutions at position R_1_–R_6_, structure-activity relationship (SAR) was studied. ‘Class A’ comprises of a cyclohexane ring substituted with the phosphate groups at positions R_1_–R_6_. Phosphate groups present at different positions of the ring have different stereo-isomeric conformations (Table 2). The hydroxyl group at position R_6_ is considered as a native group and phosphate groups at proximal positions define the IP_4_ and IP_3_ [Ins (1,4,5) P3] analogs. The native hydroxyl group appears to be more significant in interaction with the receptor or fixing the conformation of IP_3_ within the binding pocket [87]. In the present dataset, 2A is having a native hydroxyl group at position R_6_ shows high inhibitory potency (IC_50_ = 2.9 nM) against IP_3_R comparative to other compounds in the series. Similarly, 8A, 10A, 6A, 1A, and 12A also have native hydroxyl group at the position R_6,_ but the inhibitory potencies (IC_50_ = 4.43 nM, IC_50_ = 6.1 nM, IC_50_ = 14nM, IC_50_ = 26.5 nM and IC_50_ = 20,000 nM respectively) of all the compounds vary significantly. Briefly, 12A has the native hydroxyl group but has the least inhibitory potency (IC_50_ = 20,000 nM) in class A. This seems the presence of a native hydroxyl group alone is not sufficient in defining the inhibitory potencies or fixing the IP_3_ molecule within the binding pocket of IP_3_R. The phosphate groups present at other positions may also have an important role in the interaction of IP_3_ within its receptor or Ca^2+^ mobilizing activity. Previously, phosphate groups at the positions R_4_ and R_5_ were reported as significant positions in fixing the IP_3_ conformation within its receptor and defining the interactions [87].

A pair of compounds 1A (*R,S,R,S,S,S*) and 6A (*S,S,R,R,R,S*) are stereo-isomers having the biological activity 26.5 nM and 14 nM respectively. Both the compounds have a native hydroxyl group and phosphate groups at the positions R_4_ and R_5_. Two-fold higher biological activity of 6A comparative to 1A indicates that ‘*R*’ conformation of R_5_ in combination with the native hydroxyl group is more favored for IP_3_ interaction and defining the biological activity of molecule against IP_3_R. Thus, the higher biological activity values of 2A (IC_50_ = 2.9 nM), 8A (IC_50_ = 4.43 nM), and 10A (IC_50_ = 6.1 nM) could be associated to ‘*R*’ conformation of R_5_ position.

Considering this, 12A has the combination of the phosphate groups at positions R_4_ and R_5_ (*S* configuration) whereas, OH group at the native position but is least potent (IC_50_ = 20,000 nM) compared to other compounds of class A. Previously, in a study Safrany et al. have also described the important role of these positions in the binding affinity of inositol [87,88]. Hence, it is speculated that in addition to the OH group at position R_6_ and the phosphate groups at positions R_4_ and R_5_, there must be another position that plays a significant role in defining the inhibitory potency of the compounds. Therefore, the high biological activity value of 2A (IC_50_ = 2.9 nM), and 8A (IC_50_ = 4.43 nM) comparative to 12A (IC_50_ = 20,000 nM) is associated with the presence of a phosphate group at position R_1_ (*R* conformation) in combination of phosphate groups at positions R_4_ and R_5_ (*R* conformation) with a native hydroxyl group at position R_6_.

Additionally, Class B consists of stereo-isomeric derivatives of macro-cyclic bis-1-oxaquinolizidines. Compounds 1B and 3B represent a stereo-isomeric pair with the difference in stereochemistry at position R_5_. Four-fold higher biological activity of 3B compared to 1B is might be linked to *R*-configuration at position R_5_. Moreover, the 1B (IC_50_ = 2535 nM) and 2B (IC_50_ = 6400 nM) shares the same stereochemistry (*S,S,R,S,S,R*), with the only difference of a hydroxyl group at position R_2_. Three-fold decrease in the inhibitory potency of 2B is might be due to low clogP value (1B/2B: 8.01/6.70). Generally, the increase in lipophilicity of the compounds in class B results in the increase of the inhibitory potency of the compounds.

### 3.3. Pose Selection and Molecular Docking Simulations

**Figure 3:**
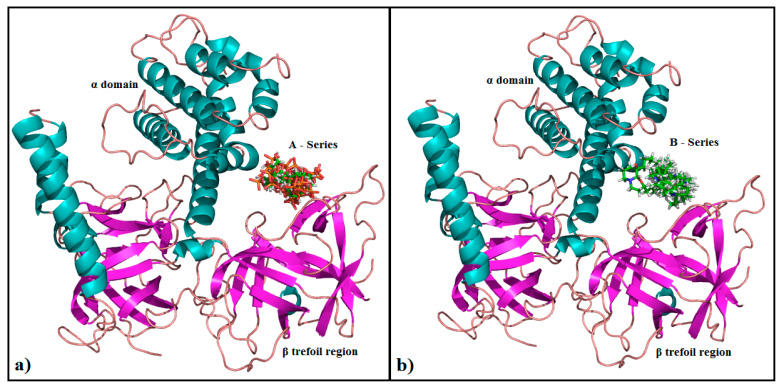
IP_3_R binding core showing α domain in cyan and β trefoil region in magentas. (**a**). The orientation of docking solutions of compounds in class ‘A’, clustered in the binding pocket of IP_3_R. (**b**). The orientation of docking solutions of compounds in class ‘B’, clustered in the binding pocket of IP_3_R.

The top--cored Alpha HB scoring function in MOE v2019.01) poses of each ligand was selected for further ligand-protein interaction analysis using PyMOL (2.0.2) molecular graphics system [93] software. The correlation plot between the binding energy scores and the pIC_50_ values is provided in Supplementary Figure S1. Overall, compounds data in both series A and B are positioned between β trefoil region and α domain in IP_3_R binding core (Figure 3a,b) and showed interaction with the conserved arginine and lysine residues of β trefoil region and α domain as mentioned in previous studies [94,95].

**Figure 4:**
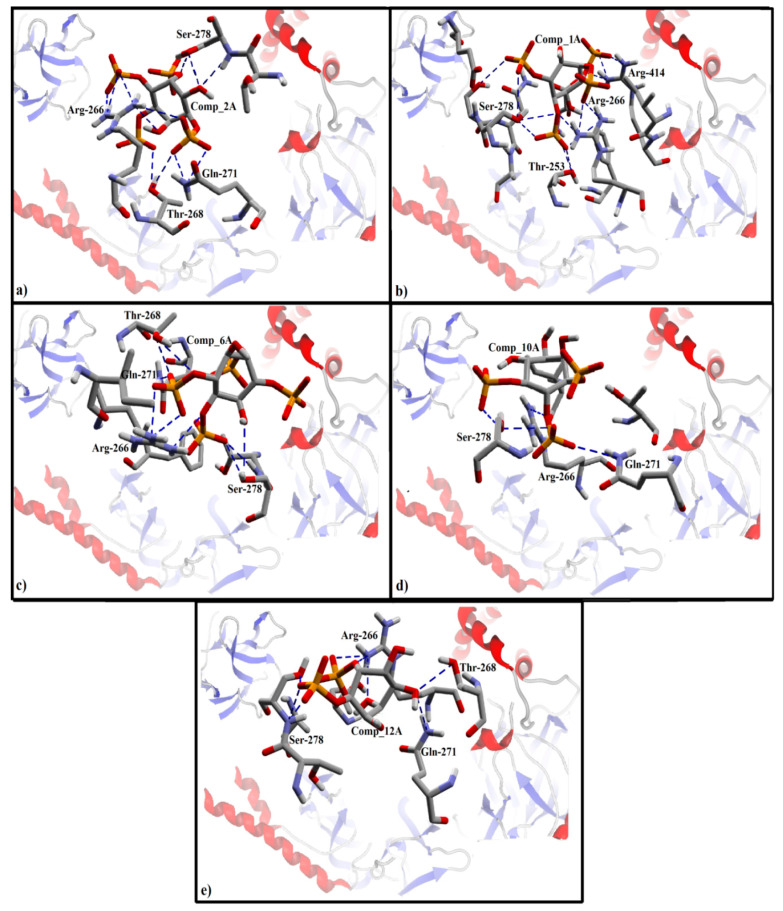
The ligand-protein interactions of docking solutions of class A in the binding pocket of IP_3_R. The backbone of protein is represented as a secondary structure. The interacting residues are shown in stick form with elements colors scheme. (**a**). Finally selected docked pose of 2Ain the binding pocket of IP_3_R showing hydrogen bond acceptor interactions with Arg-266, Thr-268, and Ser-278. (**b**). The final docked pose of 1A in the binding pocket of IP_3_R showing hydrogen bond interactions with Arg-266, Thr-268, Ser-278, and Arg-414. (**c**). The docked pose of 6A in the binding pocket of IP_3_R is showing hydrogen bond interactions with Arg-266, Thr-268, and Ser-278. (**d**). Final docked pose of 10A in the binding pocket of IP_3_R showing hydrogen bond interactions with Arg-266, and Ser-278. (**e**). Final docked pose of12A in the binding pocket of IP_3_R showing hydrogen bond interaction with Arg-266, Thr-268, and Ser-278.

In addition to the R_4_, R_5_ phosphate groups and a native hydroxyl group, the phosphate group present at R_1_ (*R* conformation) position has a significant role in defining the biological activity of the compounds, as proposed by SAR analysis of series A. 2A being the most potent (IC_50_ = 2.9 nM) compound in the class ‘A’ showed hydrogen bond interactions in addition to surface contacts and π–π interactions. Briefly, R_1_ (*R* conformation) is involved in hydrogen bond acceptor interactions with the side chain of Arg-266, Thr-268, and Gln-271 residues. A surface contact (maybe π–π interaction) was also found between the R_1_ and Arg-266 (Figure 4a). Similarly, in stereo-isomeric pair (1A and 6A), R_1_ (*R* conformation) of 1A showed hydrogen bond acceptor interaction with the side chain of Thr-253, Arg-266, and Ser-278 (Figure 4b). Taking into account the role of ‘*R’* conformation of R_1_ position, no interactions were observed at R_1_ (*S* conformation) position of 6A (Figure 4c), which further supports the importance of ‘*R*’-conformation of R_1_ in the binding of IP_3_ analogs.

Furthermore, phosphate group present at the positions R_4_ and R_5_ are considered important in IP_3_ binding with its receptor as described in different studies [49,87]. Despite the difference in stereochemistry, the R_4_ phosphate group of all compounds either most active or least active interacted with the receptor in a favored manner. For instance, R_4_ (*S* conformation) of 2A and 1A formed a hydrogen bond acceptor interaction with the side chain of Arg-266 residue (Figure 4a,b). In contrast, R_4_ (*R* conformation) of 6A formed multiple side chain hydrogen bond interactions with Arg-266, Thr-268, and Gln-271 in addition to a surface contact or π–π interaction with Arg-266 (Figure 4c). Further, the R_4_ (*S* conformation) phosphate group of 10A showed hydrogen bond donor interaction with Ser-278 (Figure 4d). Also, in 12A, the R_4_ (*R* conformation) interacts with the side chain of Arg-266 residue (Figure 4e). Thus, it seems that stereochemistry at position R_4_ is not associated with differences in biological activities of the data.

Nevertheless, R_5_ (*R* conformation) in 2A showed hydrogen bond interaction with Ser-278 (Figure 4a). Similarly, a side chain hydrogen bond interaction was observed between the R_5_ (*R* conformation) of 6A and Ser-278 residue (Figure 4c). In addition to hydrophobic or π–π interactions, R_5_ (*R* configuration) of 10A showed hydrogen bond interaction with the side chain of Arg-266, Gln-271, and Ser-278 residues (Figure 4d). Comparatively, due to difference of conformation at position R_5_ in 1A and 12A, a side chain hydrogen bond interaction was observed between R_5_ (*S* conformation) of 1A and Arg-414 residue (Figure 4b). Whereas, in 12A, R_5_ (*S* configuration) showed one hydrogen bond interaction with Ser-278 (Figure 4e). The multiple interactions of ‘*R*’ conformation of R_5_ phosphate group in 2A, 6A, and 10A as compared to 12A could be associated with the high biological activity of 2A, 6A, and 10A. Moreover, the R_5_ phosphate group of compounds 2A, 6A, and 10A specifically interacts with the Ser-278 residue indicating that it might be an important residue in the binding pocket of IP_3_R.

Likewise, the native hydroxyl group (R_6_) is considered crucial in defining the inhibitory potency of IP_3_ and in binding with IP_3_R. A decrease in inhibitory potency was observed in 4A when the native hydroxyl group was replaced with a phosphate group at the position R_6_ (Table 2). The R_6_ (OH group) of 2A showed a strong hydrogen bond interaction with Arg-266 (Figure 4a). Similarly, a strong hydrogen bond donor interaction has been observed between the R_6_ (OH group) of 1A and Ser-278 residue (Figure 4b).

**Figure 5:**
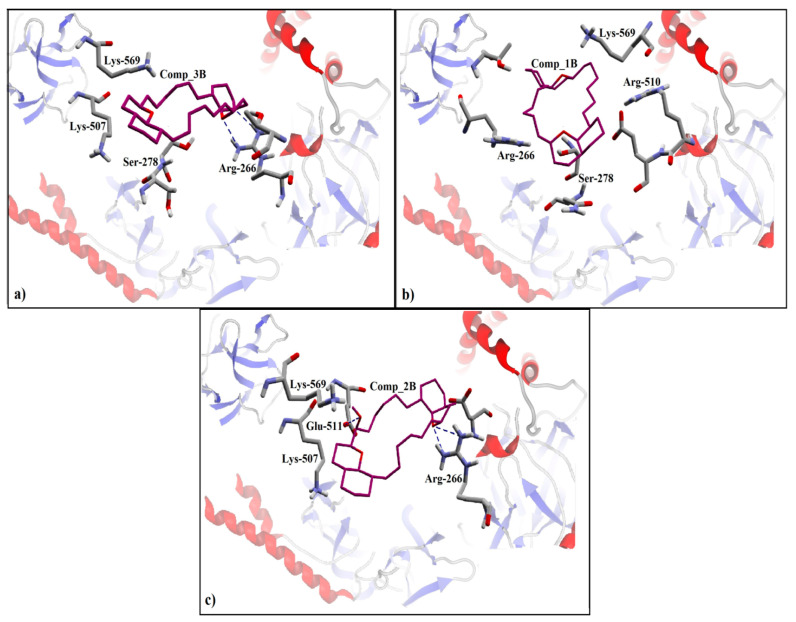
The ligand protein interactions of docking solutions of Class B in the binding pocket of IP_3_R. The back bone of protein is represented as a secondary structure. The interacting residues are shown in stick form with elements colors scheme. (**a**) The hydrogen bond interactions of a final docked pose of 3B with Arg-266. (**b**) Final docked pose of 1B showing the π–π interaction with Lys-507, and hydrophobic interaction (surface contact) with Arg-266. (**c**) 2B showing hydrogen bond interaction with Arg-266, and Glu-511.

In general, due to ring structure, the compounds of class ‘B’ were found to be involved in hydrophobic or π–π interactions within the binding pocket of IP_3_R. Among the stereo isomeric pair (1B and 3B) of class ‘B’, the R_5_ (*R* conformation) of 3B in the ring structure of oxaquinolizidine formed a hydrophobic interaction (π–π interactions) with Lys-507 and Lys-569 in addition to a side chain hydrogen bond acceptor interaction with Arg-266 residue (Figure 5a). Furthermore, the ring structure of oxaquinolizidine in 1B formed a π–π interaction with Lys-569 (Figure 5b). Similarly, 2B is the hydroxyl derivative of 1B, where the hydroxyl group present at the position R_2_ formed a hydrogen bond acceptor interaction with Glu-511. Additionally, a side chain hydrogen bond acceptor interaction was observed between the ring structure of oxaquinolizidine and Arg-266 residue (Figure 5c).

### 3.4. Protein Ligand Interaction Fingerprints (PLIF) Analysis

The interaction patterns (like surface contacts, hydrogen bonds and ionic interactions) between the IP_3_R protein [PDB ID: 6DQJ] and ligands dataset were summarized by using a fingerprint scheme of the Protein-Ligand Interaction Fingerprints (PLIF) tool in MOE 2019.01 [54]. The occurrence frequency of interactions between ligand dataset and IP_3_R is represented in the form of a population histogram. In PLIF analysis, usually residues may involve in surface interactions, ionic interactions, and hydrogen bond (acceptor or donor) interactions with either side chains or backbone. Where the H bond is calculated by contact statistics of protein and the scores are assigned on the basis of distances between the pair of atoms and their orientation. Whereby, the goodness of hydrogen bond in percentage probability is represented in the form of scores [96]. Our PLIF analysis of ligand dataset revealed the presence of hydrogen bond donor and acceptor (HBD and HBA) interactions and π–π interactions with Arg-266, Arg-510, and Ser-278 while hydrophobic and surface contacts with Arg-266, Thr-268, Ser-278, Lys-507, and Tyr-569 backbone and side chain amino acid residues. Overall, 95% of ligands in the docking conformations showed π–π interaction with residue Arg-266 side chain, while 63% of total data of poses showed direct hydrogen bond acceptor interaction with Arg-266. However, 40% of the ligands poses showed backbone and side chain hydrogen bond acceptor interactions with Ser-278.

**Figure 6:**
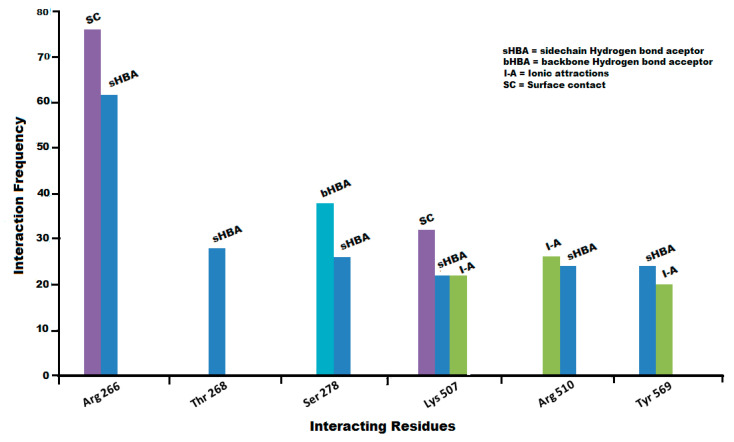
The summarized population histogram showing the frequency of occurrence of interactions profiling between residues of ligands dataset and IP_3_R protein. Arg-266 involves in the side chain hydrogen bond acceptor interactions and surface contacts with the receptor (IP_3_R) protein. Similarly, Ser-278 and Lys-507 form hydrogen bond acceptor interactions and surface contacts with the receptor (IP_3_R) protein.

Previously, site-directed mutagenic studies conducted in rats revealed that the IP_3_ binding domain comprises of conserved lysine and arginine residues playing a critical role in IP_3_ (ligand) binding, wherein the Arg-265, Lys-508, and Arg-510 were most important [97]. This further strengthens our docking outcomes. Additionally, Arg-266 (π–π interaction, surface contacts, and hydrogen bond acceptor interactions), Ser-278 (hydrogen bond acceptor interactions), and Lys-507 (hydrogen bond acceptor interactions, and surface contact) were found to be most interactive residue in almost all docked poses (Figure 6).

## 4. Discussion

In cancer cells, the IP_3_R mediated calcium signaling mechanism undergoes extensive changes in terms of activation or inhibition of oncogenes or tumor suppressors, favoring the process of oncogenesis [98,99,100,101]. Recently, the remodeling of IP_3_R mediated calcium signaling in tumorigenesis has been documented in different studies [102,103].

Herein, a knowledge-driven biological regulatory network (BRN) has been constructed to highlight the role of oncogenes and tumor suppressor genes interaction with IP_3_R in cell cycle progression, proliferation, and apoptosis. We have simulated the BRN and observed that the IP_3_R node has a central role in the network. Where the silencing of IP_3_R node (by setting its value 0.0, i.e., inactive throughout the entire simulation) results in the termination of simulations without generating any signal peaks. So, this node is established as a stable state (SS) node prior to simulations.

Our simulation results demonstrate that IP_3_R activates calcium signaling that scrutinizes CALM and CAMK-II cascades for cell proliferation and cell migration (Supplementary Figure S2). The constructed BRN and simulation results are supported by previous studies [21,104,105,106] where IP_3_R mediated Ca^+2^ signaling and Ca^+2^ dependent proteins with downstream signaling pathways are considered important in initiating the cell cycle progression. Conversely, some studies also showed the importance of regulation of Ca^+2^ mediated secretory pathways in cancer cell migration [107,108]. In this context and based upon previous studies showing the importance of IP_3_R mediated Ca^+2^ signaling, we have studied the underlying molecular pathway by constructing a BRN and simulating the IP_3_R mediatedCa^+2^ signaling. Our study demonstrates that the Ca^+2^ signaling mediated by IP_3_R recruits other binding proteins towards IP_3_R (described in other studies as well) [104,105], thereby initiating the downstream cell proliferative and apoptotic signaling cascades. We were able to produce oscillatory signals of IP_3_R by addition of perturbation function, to estimate the effect and difference of IP_3_R inhibition in normal and cancer cells. It has been observed that in normal cells, the interruption of IP_3_R signal is leading towards the inscrutable calcium signals (oscillation) creating stress and activating pro-survival response (i.e., autophagy) instead of apoptosis. In contrast, the inhibition of IP_3_R in tumorigenic cells causes insufficient transfer of Ca^2+^ signals that halts the cell cycle progression, resulting in a low cell proliferation, and hence are arrested by initiating the apoptosis. Similarly, an increased apoptotic flux is observed in tumorigenic cells, when IP_3_Rs are inhibited by external pharmacological inhibitors (Supplementary Figure S2). Previous studies [12,71,109] demonstrated that the cell survival response (autophagy) may be detrimental for cell death if the attenuation of IP_3_R continued for a prolonged time and IP_3_R fail to sustain a normal functional pool. Failure of the IP_3_R to redeem its normal function is leading towards the intrinsic apoptotic pathway initiated by induction of MOMP stimuli [72,73,110], which in turn mediates BCL-2 family along with BAX and BAK [73,111,112]. The manipulation of Ca^2+^ signaling from the endoplasmic reticulum to mitochondria via members of the Bcl-2/Bax family is considered a crucial step in the regulation of cell death [66,67].

Additionally, our SAR analysis shows that the stereo-specificity has an important role in defining the inhibitory potency of IP_3_ molecule and analogs in addition to binding interactions with its receptor. We found that although phosphates groups present at position R_4_ and R_5_ are important in defining the inhibitory potency of a compound, specifically, when an R_5_ phosphate group is present in *‘R*’-configuration. Previously, the importance of this position has been described in many studies [87,88] but we found the most favored conformation of R_5_ phosphate group via structure-activity relationship (SAR) and molecular docking studies. Our docking results further validate this hypothesis by indicating that the R_5_ not only forms hydrogen bond interactions with its receptor but also have π–π interactions. Early studies about IP_3_R [113,114,115] demonstrated that the IP_3_ molecule mostly interacts with its receptor in stereospecific manner, where R_4_ and R_5_ vicinal phosphate groups are considered as a pivotal structural feature in defining binding specificity [115]. Recently, the findings of Mills et al. [49] also prove the importance of these positions in Ca^+2^ release activity.

Furthermore, our results show that in a combination with R_5,_ the native hydroxyl group (*S* configuration) is the most favored one in binding. Replacement of native OH group with the phosphate group results in a tremendous decrease of inhibitory activity. Hence, the hydroxyl group at a native position is considered an essential group [116]. Additionally, our SAR study suggests that the phosphate group present at R_1_ position also has a significant role in defining the inhibitory potency. Previous studies [115] revealed that the phosphate group present at position R_1_ contributes towards the specific binding with receptor. Moreover, the phosphate group at position R_1_ is considered an important group to improve the IP_3_ binding affinity with its receptor [116]. Recently, Mills and coworkers [49] found that the deletion of phosphate group from this position may result in the reduction of inhibitory potency. Similarly, bis-1-oxaquinolizidines Xestospongin derivatives are considered to provide an ideal lipophilic interaction within the binding pocket of IP_3_R. The addition of a hydrogen bond donor at position R_5_ of oxaquinolizidines ring structure is associated with the decrease of potency by two-folds [90]. Our docking results further support these interactions.

Moreover, we also found that the IP_3_ molecule, when bound within the pocket, is mostly surrounded by positively charged residues i.e., Arg-266, Arg-270 from α domain, and Arg-503, Lys-507, Arg-510, and Lys 569 from the β trefoil region. Herein, our ligand–protein interaction profiling (PLIF) and molecular docking simulation results suggest that Arg-266 is the most active residue found in almost all binding solution of each ligand, forming hydrogen bond acceptor interactions in addition to surface contacts, and π–π interactions. The Arg-503 and Arg-510 actively participate in the protein-ligand interaction forming side-chain hydrogen bond acceptor interactions, and surface contacts including hydrophobic, and π–π interactions. These findings are supported by previously reported site-directed mutagenesis [97,117] studies coupled with recent molecular structural-based studies [95,118] of IP_3_R, that conserved residues, particularly Arg and Lys, are crucial for ligand binding. Moreover, taking into account the ligand–protein interaction profiling (PLIF) and molecular docking analysis, we propose that Ser-278 residue forming side chain HBA and HBD interactions in addition to backbone HBD interactions may have a crucial role in the binding of ligands, particularly antagonists of IP_3_R.

## 5. Conclusions

Our finding highlights the therapeutic importance of IP_3_R mediated calcium signaling and its inhibition in cancer cells. In order to prevent metastasis in cancer cells, targeting calcium signaling is challenging, as prevention of Ca^+2^ signaling is likely to have an impact on normal cells as well. In this study, a knowledge-driven pathway is being simulated in order to understand the downstream effectors of IP_3_R mediated Ca^+2^ signaling in the cell proliferation and apoptosis. In order to develop anti-cancer drugs against calcium signaling tool kit targets, IP_3_R has been recognized as a therapeutic hub to target through deregulation of IP_3_R mediated Ca^+2^ signaling and by introducing small molecules (inhibitors). Overall, our SAR analysis revealed the stereospecific importance of R_5_ position in the binding of IP_3_ derivatives and similar compounds. This may further lead towards the virtual screening and synthesis of more potent and stereospecific analogs of inhibitors for IP_3_R deregulation. Moreover, Arg-266 and Ser-278 are found as crucial residues in the binding of antagonists with IP_3_R. Collectively, this study not only highlights the role of IP_3_R and underlying molecular mechanism involved in cancer cell proliferation, but also sheds light upon the presence of the inhibitor and its effect on the induction of apoptosis in a cancer cell. Moreover, the binding hypothesis may provide a more precise way to target IP_3_R deregulation by introducing specific inhibitors.

## Figures and Tables

**Table 1:** Parameter values of onco- & proto-oncogenes interacting with IP_3_R before and after simulations generated in Jimena.

Nodes	In Normal Cell				In Cancer Cell			
	without IP_3_R Perturbation		with IP_3_R Perturbation		without Inhibitor		with Inhibitor	
	before Simulation	after Simulation	before Simulation	after Simulation	before Simulation	after Simulation	before Simulation	after Simulation
IP3R	0.0	0.945	0.5 *	0.393	1.0	1.0	1.0 *	0.949
Ca^+2^	0.0	0.926	0.0	0.333	0.0	0.99	0.9	0.951
CALM	0.0	0.929	0.0	0.521	0.0	1.0	0.0	0.344
CAMK-II	0.0	0.929	0.0	0.521	0.0	1.0	0.0	0.33
Proliferation	0.0	0.956	0.0	0.141	0.0	1.0	0.0	0.148
Migration	0.0	0.931	0.0	0.383	0.0	1.0	0.0	0.193
BAD	0.0	0.192	0.0	0.994	0.0	0.0	0.0	0.892
BAX	0.0	0.0	0.0	0.93	0.0	0.0	0.0	0.928
MOMP	0.0	0.0	0.0	0.997	0.0	0.0	0.0	0.997
Cyto.C	0.0	0.0	0.0	1.0	0.0	0.0	0.0	1.0
Apoptosis	0.0	0.0	0.0	1.0	0.0	0.0	0.0	1.0
Inhibitors	--	--	--	--	--	--	1.0	0.0

* Perturbation function added to the node IP_3_R and Ca^+2^.

**Table 2:** The position specific substitutions and the stereochemistry of the compounds in class A and class B.

**Inositol Phosphate (IP)** **(Class A)**			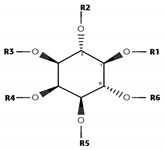							
**No.**	**Molecule**	**Conformation**	**Positions**						**IC_50_** **nM**	**Ref.**
			**R_1_**	**R_2_**	**R_3_**	**R_4_**	**R_5_**	**R_6_**		
1A	DL-Ins(1,2,4,5)P4	(R,S,R,S,S,S)	PO_3_^−2^	PO_3_^−2^	OH	PO_3_^−2^	PO_3_^−2^	OH	26.5	[48]
2A	Ins(1,3,4,5)P4	(R,S,S,S,R,S)	PO_3_^−2^	OH	PO_3_^−2^	PO_3_^−2^	PO_3_^−2^	OH	2.9	[47]
3A	Ins(1,4,5,6)P4	(R,S,S,R,R,S)	PO_3_^−2^	OH	OH	PO_3_^−2^	PO_3_^−2^	PO_3_^−2^	430	[47]
4A	Ins(3,4,5,6)P4	(S,R,R,S,R,R)	OH	OH	PO_3_^−2^	PO_3_^−2^	PO_3_^−2^	PO_3_^−2^	620	[47]
5A	D-chiro-Ins(1,3,4,6)P4	(R,S,R,S,S,R)	PO_3_^−2^	OH	PO_3_^−2^	PO_3_^−2^	OH	PO_3_^−2^	172	[48]
6A	scyllo-Ins(1,2,4,5)P4	(S,S,R,R,R,S)	PO_3_^−2^	PO_3_^−2^	OH	PO_3_^−2^	PO_3_^−2^	OH	14	[48]
7A	DL-scyllo-Ins(1,2,4)P3	(S,S,R,R,R,R)	PO_3_^−2^	PO_3_^−2^	OH	PO_3_^−2^	OH	OH	52	[48]
8A	Ins(1,4,5)P3	(R,R,S,R,R,S)	PO_3_^−2^	OH	OH	PO_3_^−2^	PO_3_^−2^	OH	4.43	[48]
9A	Ins(1,5,6)P3	(R,R,S,R,R,S)	PO_3_^−2^	OH	OH	OH	PO_3_^−2^	PO_3_^−2^	44	[47]
10A	Ins(3,4,5)P3	(R,S,S,S,R,S)	OH	OH	PO_3_^−2^	PO_3_^−2^	PO_3_^−2^	OH	6.1	[47]
11A	Ins(4,5,6)P3	(R,R,S,S,R,S)	OH	OH	OH	PO_3_^−2^	PO_3_^−2^	PO_3_^−2^	93,000	[89]
12A	Ins(4, 5)P2	(R,R,S,R,S,S)	OH	OH	OH	PO_3_^−2^	PO_3_^−2^	OH	20,000	[89]
**Xestospongins (Xe)** **(Class B)**			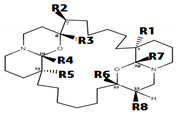							
**No.**	**Molecule**	**Conformation**	**Positions**				**IC_50_** **nM**		**clogP**	**Ref.**
			**R_1_**	**R_2_**	**R_5_**	**R_8_**				
			**C1**	**C7**	**C15**	**C32**				
1B	Xestospongin_A	1S,8S,10R,15S,22S,29R	---	---	---	---	2535		8.01	[90]
2B	7-(OH)-XeA	1S,7S,8R,10R,15S,22S,29R	---	OH	---	---	6400		6.70	[91]
3B	Araguspongine B	1R,8S,10R,15R,22S,29R	---	--	---	---	648		8.01	[90]
4B	Araguspongine C	1R,8R,10S,15R,22R,29S	OH	---	OH	---	6600		4.69	[91]
5B	Xestospongin_B	1S,8S,10R,15R,22S,29R,32R	---	---	OH	CH_3_	5000		7.29	[92]
6B	De Methylated XeB	1S,8S,10R,15R,22S,29R	---	--	OH	----	5865		6.77	[90]

## Data Availability

The data presented in this study is available in supplementary material.

## Supplementary Materials

The following are available online at https://www.mdpi.com/2073-4425/12/1/34/s1, Supplementary File_IP3R inhibitors.xlsx; and Supplementary Material _Biological Network Analysis.doc. Figure S1: “Correlation plot of the binding poses of the data set of IP3R modulators showing better correlation (R2) between binding energy score and pIC50 value”; Figure S2: “Dynamic simulation of biological regulatory network. The sequence of events in case of proliferation & apoptosis in normal and cancer cell”; Table S1: “Onco-proteins and tumor suppressor proteins interacting with IP3R”.
