# Supplementary material for: Biological Regulatory Network (BRN) Analysis and Molecular Docking Simulations to Probe the Modulation of IP3R Mediated Ca2+ Signaling in Cancer
Source: Genes (Basel). 2020 Dec 29;12(1):34. doi: 10.3390/genes12010034 (PMC7823498; doi:10.3390/genes12010034)
Supplement: Supplementary file 1 [file genes-12-00034-s001.zip › Supplementary Material_Biological Regulatory Network.docx]

**Biological Regulatory Network (BRN) Analysis and Molecular Docking Simulations to Probe the Modulation of IP_3_R Mediated Ca^2+^ Signaling in Cancer**

Humaira Ismatullah, Ishrat Jabeen* and Muhammad Tariq Saeed

Research Center for Modeling and Simulation (RCMS), National University of Sciences and Technology (NUST), Academic-I Building, H-12 Islamabad, Pakistan

*Corresponding Author: ​ishrat.jabeen​@rcms.nust.edu.pk

**Supplementary Material**

**Contents**

**Materials and Methods**

| **Table S1** | **Onco-proteins and tumor suppressor proteins interacting with IP_3_R** |
| --- | --- |

**Results & Discussion**

| **Figure S1**  **Figure S2** | **Correlation plot of the binding poses of the data set of IP_3_R modulators showing better correlation (R^2^) between binding energy score and pIC_50_ value**  **Dynamic simulation of biological regulatory network. The sequence of events in case of proliferation & apoptosis in normal and cancer cell** |
| --- | --- |

1. **Materials and Methods:**

In order to construct a biological regulatory network (BRN) we refined and build the topology of available information about interactive data from literature [[1-6](#_ENREF_1)] and Kyoto Encyclopedia of Genes and Genomes (KEGG) databank. The interactive oncogenes and proto oncogenes are summarized in supplementary **Table S1**.

**Table S1: Onco-proteins and tumor suppressor proteins interacting with IP_3_R**

| **Protein** | **Function** | **Ref** |
| --- | --- | --- |
| AKT | Phosphorylate IP_3_Rs and have negative effect on Ca^2+^ dependent apoptosis. | [[7-10](#_ENREF_7)] |
| mTOR | Controls phosphorylation of IP_3_R regulated by AKT and promotes Ca^2+^ uptake. | [[9](#_ENREF_9), [11](#_ENREF_11), [12](#_ENREF_12)] |
| PTEN | De phosphorylates IP3Rs and Sensitizes cells to Ca^2+^ dependent apoptosis. | [[2](#_ENREF_2), [13](#_ENREF_13), [14](#_ENREF_14)] |
| PML | Forms a complex with AKT and PP2A and promotes ER-mitochondria Ca^2+^ transfer and apoptosis. PP2A suppresses AKT-mediated IP_3_R_3_ phosphorylation. | [[15](#_ENREF_15), [16](#_ENREF_16)] |
| p53 | It regulates Ca2+ release in IP_3_R_3_ via interacting with SERCA pumps. | [[17](#_ENREF_17), [18](#_ENREF_18)] |
| Bcl-2 | It limits the transfer of pro-apoptotic Ca^2+^ signals and can induce leakage of Ca^2+^ from ER to the mitochondria by acting on both organelles. | [[19-21](#_ENREF_19)] |
| Bcl-X_L_ | Enhances Ca^2+^ oscillations and increases basal IP_3_R function. When IP3 level lower down it sensitizing the channels to IP_3_R, thus act as pro-survival protein. | [[22](#_ENREF_22), [23](#_ENREF_23)] |
| BAD | It binds with Bcl-X_L_ when dephosphorylated and antagonizes its anti- apoptotic effect. Thus activates apoptosis by subsequent activation of Bax/Bak. | [[24](#_ENREF_24), [25](#_ENREF_25)] |
| CaN | Ca^2+^ signals activates CaN that dephosphorylates NFAT. This leads NFAT to translocate into the nucleus and transcription activated. It involves in cell cycle progression. | [[26-30](#_ENREF_26)] |
| CaMKII | Involves in Ca2+ signaling pathways by interacting with Filamin and involved in cell cycle progression. | [[28](#_ENREF_28), [29](#_ENREF_29), [31](#_ENREF_31)] |

1. **Results & Discussion:**


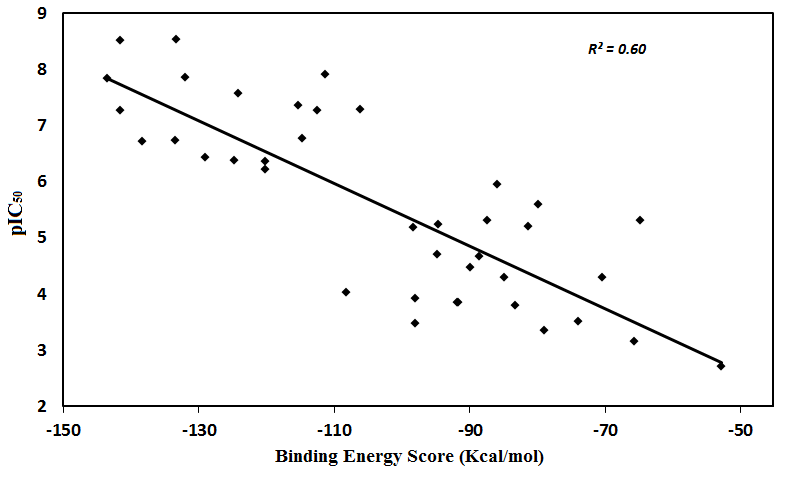


**Figure S1:** Correlation plot of the binding poses of dataset of IP_3_R modulators showing better correlation (R^2^) between binding energy score and pIC_50_ value.

1. **Biological Regulatory Network (BRN):**

IP_3_R mediated Ca^2+^ siganling is responsible for cell proliferation and apoptosis. In proliferation, different binding proteins are congregated towards the IP_3_R by Calmodulin (CaM) [[32](#_ENREF_32)], initiating the downstream signaling cascade of Ca^2+^/CaM-dependent phosphatase and Ca^2+^/CaM-kinases (CaMK) [[33](#_ENREF_33)]. Furthermore, Calcineurin (CaN), upon activation by Ca^2+^, triggers the nuclear factor of activated T-cells (NFAT) pathway [[30](#_ENREF_30), [34](#_ENREF_34)] that also has a significant role in initiation of proliferation by progressing cell cycle phases [[35-37](#_ENREF_35)]. Any disruption in IP_3_R mediated Ca^+2^ level creates stress and activates pro-survival response (i.e. autophagy) [[38](#_ENREF_38), [39](#_ENREF_39)]. However, these cell survival responses (autophagy) may lead towards the cell death if intureption prolonged and IP_3_R fails to regain its normal function. Successively, the intrinsic apoptotic pathway initiated in response, that is provoked by the mitochondrial outer membrane permeabilization (MOMP) induction [[40](#_ENREF_40), [41](#_ENREF_41)]. This induction regulates the multi domain pro-apoptotic protein family BCL-2 along with BAX and BAK [[40](#_ENREF_40), [42](#_ENREF_42), [43](#_ENREF_43)], which is considered crucial in regulation of cell death [[44](#_ENREF_44), [45](#_ENREF_45)].

Conversaly, an authoritative long term growth signal response is seen in cancer cells by reprogramming of host energy metabolism [[46](#_ENREF_46), [47](#_ENREF_47)]. In many cancer types, Bcl-2 targets the central modulatory domain of IP_3_R, thus arrests the IP_3_R activity in pro-apoptotic Ca^2+^ release events [[25](#_ENREF_25), [48](#_ENREF_48)]. Whereby, overexpression of Bcl-2 results in reduction of Ca^2+^ level within ER followed by reduced fragmentation in mitochondria that can initiate the apoptosis [[49](#_ENREF_49)]. Furthermore, tumor suppressors like PTEN and PML, and oncogenes like Akt play an important role in development of cancer via modulation of IP_3_R dependent Ca^+2^ signaling [[47](#_ENREF_47), [50](#_ENREF_50)].


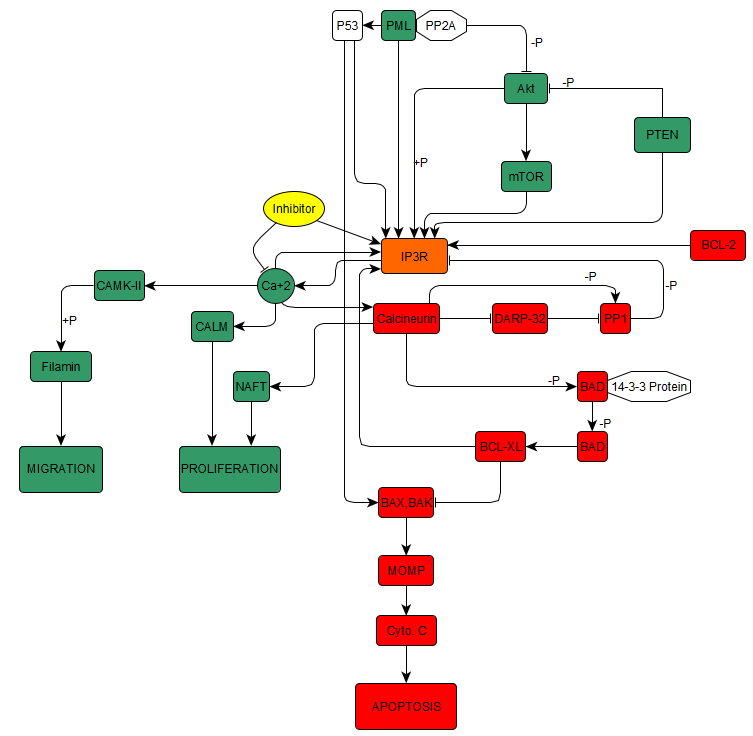


**Figure S2:** Dynamic simulation of biological regulatroy network (BRN) showing the role of IP_3_R and calcium in proliferatiion and apoptosis signaling casecade. The entities with green color shows the proliferation in normal cells. In normal cells, the proliferation pathway activates and apoptosis siganls remains at minimal level of expression. While the entities with red color shows the apoptosis when inhibitor is added in the BRN. Here, if a stress is created due to insufficient calcium pool or an ihibitor added to normal or cancerous cell that modulates the IP_3_R activity, as a result, the apoptosis induces by reducing the proliferation signal peaks in BRN.

**References:**

1. Akl, H. and G. Bultynck, *Altered Ca2+ signaling in cancer cells: proto-oncogenes and tumor suppressors targeting IP3 receptors.* Biochimica et Biophysica Acta (BBA)-Reviews on Cancer, 2013. **1835**(2): p. 180-193.

2. Song, M.S., L. Salmena, and P.P. Pandolfi, *The functions and regulation of the PTEN tumour suppressor.* Nature reviews Molecular cell biology, 2012. **13**(5): p. 283.

3. Hers, I., E.E. Vincent, and J.M. Tavaré, *Akt signalling in health and disease.* Cellular signalling, 2011. **23**(10): p. 1515-1527.

4. Stephens, L., et al., *Protein kinase B kinases that mediate phosphatidylinositol 3, 4, 5-trisphosphate-dependent activation of protein kinase B.* Science, 1998. **279**(5351): p. 710-714.

5. Oren, M., *Decision making by p53: life, death and cancer.* Cell death and differentiation, 2003. **10**(4): p. 431.

6. Laplante, M. and D.M. Sabatini, *mTOR signaling in growth control and disease.* Cell, 2012. **149**(2): p. 274-293.

7. Szado, T., et al., *Phosphorylation of inositol 1, 4, 5-trisphosphate receptors by protein kinase B/Akt inhibits Ca2+ release and apoptosis.* Proceedings of the National Academy of Sciences, 2008. **105**(7): p. 2427-2432.

8. Khan, M.T., et al., *Akt kinase phosphorylation of inositol 1, 4, 5-trisphosphate receptors.* Journal of Biological Chemistry, 2006. **281**(6): p. 3731-3737.

9. Marchi, S., et al., *Selective modulation of subtype III IP 3 R by Akt regulates ER Ca 2+ release and apoptosis.* Cell death & disease, 2012. **3**(5): p. e304.

10. Marchi, S., et al., *Akt kinase reducing endoplasmic reticulum Ca2+ release protects cells from Ca2+-dependent apoptotic stimuli.* Biochemical and biophysical research communications, 2008. **375**(4): p. 501-505.

11. Betz, C., et al., *mTOR complex 2-Akt signaling at mitochondria-associated endoplasmic reticulum membranes (MAM) regulates mitochondrial physiology.* Proceedings of the National Academy of Sciences, 2013. **110**(31): p. 12526-12534.

12. Hagiwara, A., et al., *Hepatic mTORC2 activates glycolysis and lipogenesis through Akt, glucokinase, and SREBP1c.* Cell metabolism, 2012. **15**(5): p. 725-738.

13. Bononi, A., et al., *Identification of PTEN at the ER and MAMs and its regulation of Ca 2+ signaling and apoptosis in a protein phosphatase-dependent manner.* Cell death and differentiation, 2013. **20**(12): p. 1631.

14. Blanco-Aparicio, C., et al., *PTEN, more than the AKT pathway.* Carcinogenesis, 2007. **28**(7): p. 1379-1386.

15. Giorgi, C., et al., *PML regulates apoptosis at endoplasmic reticulum by modulating calcium release.* Science, 2010. **330**(6008): p. 1247-1251.

16. Missiroli, S., et al., *PML at mitochondria-associated membranes is critical for the repression of autophagy and cancer development.* Cell reports, 2016. **16**(9): p. 2415-2427.

17. Giorgi, C., et al., *p53 at the endoplasmic reticulum regulates apoptosis in a Ca2+-dependent manner.* Proceedings of the National Academy of Sciences, 2015. **112**(6): p. 1779-1784.

18. Ottolini, D., et al., *The Parkinson disease-related protein DJ-1 counteracts mitochondrial impairment induced by the tumour suppressor protein p53 by enhancing endoplasmic reticulum–mitochondria tethering.* Human molecular genetics, 2013. **22**(11): p. 2152-2168.

19. Meunier, J. and T. Hayashi, *Sigma-1 receptors regulate Bcl-2 expression by reactive oxygen species-dependent transcriptional regulation of nuclear factor κB.* Journal of Pharmacology and Experimental Therapeutics, 2010. **332**(2): p. 388-397.

20. Rong, Y.-P., et al., *Targeting Bcl-2 based on the interaction of its BH4 domain with the inositol 1, 4, 5-trisphosphate receptor.* Biochimica et Biophysica Acta (BBA)-Molecular Cell Research, 2009. **1793**(6): p. 971-978.

21. Akl, H., et al., *A dual role for the anti-apoptotic Bcl-2 protein in cancer: mitochondria versus endoplasmic reticulum.* Biochimica et Biophysica Acta (BBA)-Molecular Cell Research, 2014. **1843**(10): p. 2240-2252.

22. White, C., et al., *The endoplasmic reticulum gateway to apoptosis by Bcl-X L modulation of the InsP 3 R.* Nature cell biology, 2005. **7**(10): p. 1021.

23. Li, C., et al., *Apoptosis regulation by Bcl-xL modulation of mammalian inositol 1, 4, 5-trisphosphate receptor channel isoform gating.* Proceedings of the National Academy of Sciences, 2007. **104**(30): p. 12565-12570.

24. Wang, H.-G., et al., *Ca2+-induced apoptosis through calcineurin dephosphorylation of BAD.* Science, 1999. **284**(5412): p. 339-343.

25. Vervliet, T., J. Parys, and G. Bultynck, *Bcl-2 proteins and calcium signaling: complexity beneath the surface.* Oncogene, 2016. **35**(39): p. 5079.

26. Kawano, S., et al., *ATP autocrine/paracrine signaling induces calcium oscillations and NFAT activation in human mesenchymal stem cells.* Cell calcium, 2006. **39**(4): p. 313-324.

27. Vukcevic, M., et al., *Frequent calcium oscillations lead to NFAT activation in human immature dendritic cells.* Journal of Biological Chemistry, 2010. **285**(21): p. 16003-16011.

28. Kahl, C.R. and A.R. Means, *Regulation of cell cycle progression by calcium/calmodulin-dependent pathways.* Endocrine reviews, 2003. **24**(6): p. 719-736.

29. Pinto, M.C.X., et al., *Calcium signaling and cell proliferation.* Cellular signalling, 2015. **27**(11): p. 2139-2149.

30. Berridge, M.J., P. Lipp, and M.D. Bootman, *The versatility and universality of calcium signalling.* Nature reviews Molecular cell biology, 2000. **1**(1): p. 11.

31. Lorca, T., et al., *Calmodulin-dependent protein kinase II mediates inactivation of MPF and CSF upon fertilization of Xenopus eggs.* Nature, 1993. **366**(6452): p. 270.

32. Chin, D. and A.R. Means, *Calmodulin: a prototypical calcium sensor.* Trends in cell biology, 2000. **10**(8): p. 322-328.

33. Hofer, A.M. and K. Lefkimmiatis, *Extracellular calcium and cAMP: second messengers as “third messengers”?* Physiology, 2007. **22**(5): p. 320-327.

34. Crabtree, G.R., *Generic signals and specific outcomes: signaling through Ca2+, calcineurin, and NF-AT.* Cell, 1999. **96**(5): p. 611-614.

35. Capiod, T., *Cell proliferation, calcium influx and calcium channels.* Biochimie, 2011. **93**(12): p. 2075-2079.

36. Borowiec, A.-S., et al., *Calcium channels, external calcium concentration and cell proliferation.* European journal of pharmacology, 2014. **739**: p. 19-25.

37. Roderick, H.L. and S.J. Cook, *Ca 2+ signalling checkpoints in cancer: remodelling Ca 2+ for cancer cell proliferation and survival.* Nature Reviews Cancer, 2008. **8**(5): p. 361.

38. Bultynck, G., *Onco-IP3Rs feed cancerous cravings for mitochondrial Ca2+.* Trends in biochemical sciences, 2016. **41**(5): p. 390-393.

39. Cárdenas, C., et al., *Essential regulation of cell bioenergetics by constitutive InsP3 receptor Ca2+ transfer to mitochondria.* Cell, 2010. **142**(2): p. 270-283.

40. Cosentino, K. and A.J. García-Sáez, *Mitochondrial alterations in apoptosis.* Chemistry and physics of lipids, 2014. **181**: p. 62-75.

41. Ferri, K.F. and G. Kroemer, *Organelle-specific initiation of cell death pathways.* Nature cell biology, 2001. **3**(11): p. E255.

42. Scorrano, L., et al., *BAX and BAK regulation of endoplasmic reticulum Ca2+: a control point for apoptosis.* Science, 2003. **300**(5616): p. 135-139.

43. Jourdain, A. and J.-C. Martinou, *Mitochondrial outer-membrane permeabilization and remodelling in apoptosis.* The international journal of biochemistry & cell biology, 2009. **41**(10): p. 1884-1889.

44. Nutt, L.K., et al., *Bax and Bak promote apoptosis by modulating endoplasmic reticular and mitochondrial Ca2+ stores.* Journal of Biological Chemistry, 2002. **277**(11): p. 9219-9225.

45. Chami, M., et al., *Bcl-2 and Bax exert opposing effects on Ca2+ signaling, which do not depend on their putative pore-forming region.* Journal of Biological Chemistry, 2004. **279**(52): p. 54581-54589.

46. Hanahan, D. and R.A. Weinberg, *Hallmarks of cancer: the next generation.* Cell, 2011. **144**(5): p. 646-674.

47. Maklad, A., A. Sharma, and I. Azimi, *Calcium signaling in brain cancers: roles and therapeutic targeting.* Cancers, 2019. **11**(2): p. 145.

48. Monaco, G., et al., *Selective regulation of IP 3-receptor-mediated Ca 2+ signaling and apoptosis by the BH4 domain of Bcl-2 versus Bcl-Xl.* Cell death and differentiation, 2012. **19**(2): p. 295.

49. Pinton, P., et al., *The Ca2+ concentration of the endoplasmic reticulum is a key determinant of ceramide‐induced apoptosis: significance for the molecular mechanism of Bcl‐2 action.* The EMBO journal, 2001. **20**(11): p. 2690-2701.

50. Prevarskaya, N., et al., *Remodelling of Ca2+ transport in cancer: how it contributes to cancer hallmarks?* Philosophical Transactions of the Royal Society B: Biological Sciences, 2014. **369**(1638): p. 20130097.
